# GDF-15 is abundantly expressed in plexiform lesions in patients with pulmonary arterial hypertension and affects proliferation and apoptosis of pulmonary endothelial cells

**DOI:** 10.1186/1465-9921-12-62

**Published:** 2011-05-06

**Authors:** Nils Nickel, Danny Jonigk, Tibor Kempf, Clemens L Bockmeyer, Lavinia Maegel, Johanna Rische, Florian Laenger, Ulrich Lehmann, Clemens Sauer, Mark Greer, Tobias Welte, Marius M Hoeper, Heiko A Golpon

**Affiliations:** 1 Clinic for Pulmonary Medicine, Hannover Medical School, Carl-Neuberg-Str. 1, 30625 Hannover, Germany; 2 Institute of Pathology, Hannover Medical School, Carl-Neuberg-Str. 1, 30625 Hannover, Germany; 3 Department of Cardiology and Angiology, Hannover Medical School, Carl-Neuberg-Str. 1, 30625 Hannover, Germany

## Abstract

**Background:**

Growth-differentiation factor-15 (GDF-15) is a stress-responsive, transforming growth factor-β-related cytokine, which has recently been reported to be elevated in serum of patients with idiopathic pulmonary arterial hypertension (IPAH). The aim of the study was to examine the expression and biological roles of GDF-15 in the lung of patients with pulmonary arterial hypertension (PAH).

**Methods:**

GDF-15 expression in normal lungs and lung specimens of PAH patients were studied by real-time RT-PCR and immunohistochemistry. Using laser-assisted micro-dissection, GDF-15 expression was further analyzed within vascular compartments of PAH lungs. To elucidate the role of GDF-15 on endothelial cells, human pulmonary microvascular endothelial cells (HPMEC) were exposed to hypoxia and laminar shear stress. The effects of GDF-15 on the proliferation and cell death of HPMEC were studied using recombinant GDF-15 protein.

**Results:**

GDF-15 expression was found to be increased in lung specimens from PAH patients, com-pared to normal lungs. GDF-15 was abundantly expressed in pulmonary vascular endothelial cells with a strong signal in the core of plexiform lesions. HPMEC responded with marked upregulation of GDF-15 to hypoxia and laminar shear stress. Apoptotic cell death of HPMEC was diminished, whereas HPMEC proliferation was either increased or decreased depending of the concentration of recombinant GDF-15 protein.

**Conclusions:**

GDF-15 expression is increased in PAH lungs and appears predominantly located in vascular endothelial cells. The expression pattern as well as the observed effects on proliferation and apoptosis of pulmonary endothelial cells suggest a role of GDF-15 in the homeostasis of endothelial cells in PAH patients.

## Background

GDF-15 is a protein belonging to the TGF-beta family, which includes several proteins involved in tissue homeostasis, differentiation, remodeling and repair [1]. As a pleiotropic cytokine it is involved in the stress response program of different cell types after cellular injury. Under normal conditions, GDF-15 is only weakly expressed in most tissues [2]. However GDF-15 is strongly upregulated in disease states such as acute injury, tissue hypoxia, inflammation and oxidative stress [3-6].

In the cardiovascular system, GDF-15 is expressed in cardiomyocytes and other cell types including macrophages, endothelial cells, vascular smooth muscle cells, and adipocytes [1,7,8]. In endothelial cells (ECs) it has been shown that GDF-15 inhibits proliferation, migration and invasion in vitro and in vivo [9-11]. A recent study demonstrated that the inhibitory effect of GDF-15 on EC proliferation was only present at higher concentrations (50 ng/ml), whereas at ten times lower concentrations (5 ng/ml), GDF-15 caused endothelial cell proliferation and was proangiogenic [12]. At present little is known about the expression of GDF-15 in the lung. In situ hybridization studies in rats have revealed expression of GDF-15 in bronchial epithelial cells [1]. GDF-15 is potently induced in animal models of lung injury. Bleomycin administration in adult mice and prolonged hyperoxic exposure in neonate mice resulted in GDF-15 induction [5].

Pulmonary arterial hypertension (PAH) is a life-threatening disease characterized by a marked and sustained elevation of pulmonary artery pressure that results in right ventricular (RV) failure and death [13]. Histologically, remodeling of pulmonary arteries show various degrees of medial hypertrophy and endothelial cell growth, which ultimately lead to the obliteration of precapillary arteries [14,15]. The mechanisms resulting in pulmonary vascular remodeling are complex and incompletely understood. Several members of the TGF-β superfamily have been implicated in this process [16] while the role of GDF-15 in the pathophysiology of PAH is not clear. In a recent study we demonstrated elevated serum levels of GDF-15 in patients with idiopathic pulmonary arterial hypertension (IPAH) [17]. Furthermore, it has been shown that GDF-15 serum levels are increased in scleroderma patients with pulmonary hypertension and GDF-15 protein was predominantly located in monocytes infiltrating the lung tissue [18].

In the present study we investigated the expression of GDF-15 in human normal lungs and in lung tissue from patients with PAH. In addition, we conducted *in vitro*-studies to elucidate the possible role of GDF-15 in the pulmonary vasculature.

## Methods

### Human tissue samples

Lung tissue was obtained from 5 brain-dead organ donors and explanted lungs from 7 patients with PAH (IPAH, n = 4, congenital heart disease-associated PAH, n = 3) at the time of lung transplantation. Formalin-fixed, paraffin-embedded lung tissue specimens were obtained from the Institute of Pathology at Hannover Medical School following the guidelines of the local ethics committee. Complex vascular lesions in PAH patients were diagnosed by two experienced pathologists (FL, DJ) according to well-established histopathological criteria [19].

### Immunohistochemical staining

Formalin-fixed, paraffin-embedded sections (3 μm) of normal controls and PAH lungs were deparaffinized. The endogenous peroxidase was blocked with 3% H_2_O_2 _for 10 min. GDF-15 staining was performed using a polyclonal monospecific antibody (1:20, Rabbit anti-human HPA011191, Sigma-Aldrich, Munich, Germany) after epitope retrieval with Protease XXIV (Sigma-Aldrich, Munich, Germany, 10 min, 37°C). Primary antibody was incubated for one hour at room temperature and visualised in brown with diaminobenzidine (DAB) as substrate for horseradish peroxidase (PolyHRP detection system, Zytomed Systems, Berlin, Germany). Sections were counterstained with Hemalaun. Negative controls were performed using a rabbit IgG isotype control (Dianova, Hamburg, Germany, diluted like the primary antibody). Healthy placental tissue [20] (Additional file 1 - panel A) and prostate cancer tissue [18,21] (Additional file 1 - panel B) served as control for GDF-15 immunostaining. Exemplary staining (Additional file 2) was also performed using Goat anti-human GDF-15 IgG antibody (1:25, R&D Systems, cat. no. AF957).

### Microdissection of plexiform lesions

Formalin-fixed, paraffin-embedded (FFPE) tissue sections 5 μm were mounted on a poly-L-lysin-coated membrane fixed onto a metal frame. After standard deparaffinization and hemalaun staining, the CellCut Plus system (MMI Molecular Machines & Industries AG, Glattbrugg, Switzerland) was used for laser-assisted microdissection. Distinct anatomical lung structures (plexiform lesions, normal arteries) were isolated using a no-touch technique, essentially as described earlier by our group [22]. Approximately 850 cells were harvested from serial sections in each compartment.

### Real-time RT-PCR

Extraction of total RNA and cDNA synthesis was performed as previously described (20). Real-time RT-PCR was performed on an ABI PRISM 7700 Sequence Detector (Applied Biosystems, Foster City, CA, USA). C_T _values were calculated by normalization to the mean expression of two endogenous controls (β-GUS and β-actin) and converted into 2^-DDCT ^values. For calculation of relative expression levels, the weakest signal in the control group was set equal to one, with all other values being calculated relative to this level. The primer pair for GDF-15 (Applied Biosystems, ID: Hs00171132_m1) was: *GDF-15 *(forward: *CAC ACCGAAGACTCCAGA*, reverse: *CCGAGAGATACGCAGGT*; Amplicon size 78 bp).

### Cell culture experiments

#### Human pulmonary microvascular endothelial cells

Human pulmonary microvascular endothelial cell-line (HPMEC) clone ST1.6R (kindly pro-vided by Prof. C.J. Kirkpatrick, Institute of Pathology, Johannes-Gutenberg University of Mainz) was maintained in Earles Medium 199 and supplemented with 20% fetal calf serum, 50 μg/ml endothelial cell growth supplement, 2 mM Glutamax, sodium heparin (25 μg/ml) and 1% penicillin/streptomycin. Cells were cultured at 37°C, 5% CO_2 _and passaged 2-3 times weekly using trypsin-EDTA. The cell line was characterized earlier as endothelial cells by the presence of platelet endothelial cell adhesion molecule (PECAM, CD 31), von Willebrand factor (vWF), intercellular adhesion molecule (ICAM-1), vascular cell adhesion molecule-1 (VCAM-1) and E-selectin [23]. Previous studies have demonstrated the endothelial cell properties of the cell line [24,25].

#### Hypoxic treatment

HPMEC maintained in Earles Medium 199 and supplemented with 20% fetal calf serum was seeded in 6-well plates and grown to 70-80% confluence. Hypoxia was induced in a hypoxia incubator chamber (Billups-Rothenberg, San Diego, USA) [26] for various time periods ranging between 2-12 hours. Cell viability and cell death assays were performed 2 h after hypoxia induction.

### Shear stress exposure

Shear stress experiments were performed in a modified cone-and-plate apparatus utilized for generating defined fluid shear stresses [27], consisting of a stainless steel cone rotating over a base 6-well plate that contains plastic coverslip inserts. The entire apparatus was maintained in a 5% CO_2_/95% air humidified atmosphere thermostatically regulated at 37°C. Fluid mechanical parameters were adjusted to subject the endothelial monolayers (HPMEC) to a laminar shear stress of 5 and 15 dynes/cm^2 ^(1 dyne = 100 mN) for 6 h, which reflects physiological shear stress in major human arteries that ranges between 5-20 dyn/cm^2 ^[28]. Replicate-plated control coverslips were incubated under static conditions for the same time period.

### Assessment of cell growth

For assessment of cell viability after hypoxic treatment, HPMEC were grown to 80% conflu-ence in 96-well plates. Ten minutes before starting hypoxic treatment, various concentrations (1 ng/ml to 100 ng/ml) of GDF-15 were added to each well. Cell vitality was measured using the CellTiter 96 Aqueous One solution cell proliferation assay (Promega, Madison, USA) according to the manufacturer's protocol. Absorbance of the formazan product was measured at 490 nm (Versamax tunable microplate reader, Molecular Devices, Sunnyvale, USA) [29].

### Assessment of cell death

To induce endothelial cell death, HPMEC were exposed to hypoxia as described above. To identify endothelial cell death, double staining with Annexin-V-FLUOS (Roche, Mannheim, Germany) and propidium iodide (Sigma-Aldrich, Munich, Germany) was performed in HPMEC either in absence or presence of GDF-15 (5 ng/ml or 50 ng/ml). In addition, double staining with Hoechst-33342 and sytox green (both Invitrogen Molecular Probes, Karlsruhe, Germany) was performed as described earlier [30]. The activity of caspase-3 and 7 in HPMEC cell extracts was detected using the Apo-ONE homogenous caspase-3/7 assay (Pro-mega, Mannheim, Germany), according to the manufacturer's protocol. Fluorescence was detected at an excitation wavelength of 499 nm with emission maximum at 521 nm (Versamax tunable microplate reader, Molecular Devices, Sunnyvale, USA).

### In vitro angiogenesis assay

Endothelial cell spheroids were prepared as described by Korff et al. [31]. HPMEC were suspended in a corresponding medium containing 20% methocel-stock solution (Earles Medium 199 + 1.2% methyl-cellulose (w/v); Sigma-Aldrich, Munich, Germany). A defined number of cells were seeded in the wells of a non-adherent round-bottom 96 well plate (Greiner, Frickenhausen, Germany) to form single spheroids with a defined number of cells (750) and size within 24 h at 37°C and 5% CO_2 _in humidified atmosphere. In vitro angiogenesis in collagen gels was quantified using spheroids of HPMEC as described by Korff et al. [31].

### Western Blot Analysis

Immunoblotting was performed as described earlier [32]. Polyclonal goat anti-human GDF-15 IgG antibody (R&D Systems, cat. no. AF957) was used to determine GDF-15 expression in HPMEC. Antibodies against β-actin, Akt, and Ser473-phospho-Akt were obtained from Sigma-Aldrich (Munich, Germany) or by New England Biolabs (Ipswich, USA).

### GDF-15 Sandwich IRMA

GDF-15 protein in supernatants of HPMEC was measured using an immunoradiometric sandwich assay as described previously [33]. In these experiments a polyclonal goat anti-human GDF-15 IgG antibody (R&D Systems, cat. no. AF957) was used.

### Statistical analysis

Values are presented as mean ± SD. Gaussian distribution of the values was evaluated using the Kolmogorov-Smirnov test. Comparisons between groups were tested by Student's t-test or Mann-Whitney test where appropriate. Significances between more than two groups were determined by one-way analysis of variance (ANOVA), followed by Student-Newman-Keuls post-hoc test or by Kruskal-Wallis test where appropriate. A P value < 0.05 was considered to indicate statistical significance. Analyses were performed using SPSS16.0 and GraphPad Prism version 5.01.

## Results

### GDF-15 expression in lungs of patients with PAH

**Figure 1:**
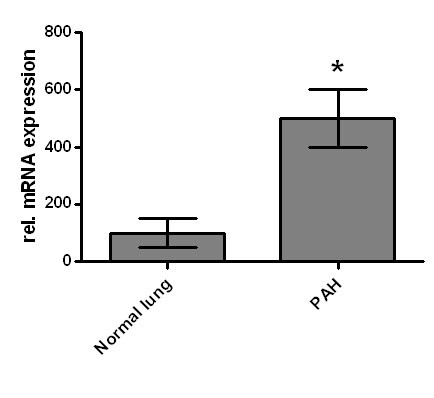
**GDF-15 mRNA expression in normal human lung**. GDF-15 mRNA expression in normal lung and lung tissue from patients with pulmonary arterial hypertension (PAH) was assessed by real-time RT-PCR. Data are presented as relative expression of GDF-15 mRNA normalized to two housekeeping genes. Data from n = 5 each group are shown as mean ± SD. * = p < 0.05 vs. normal lung.

**Figure 2:**
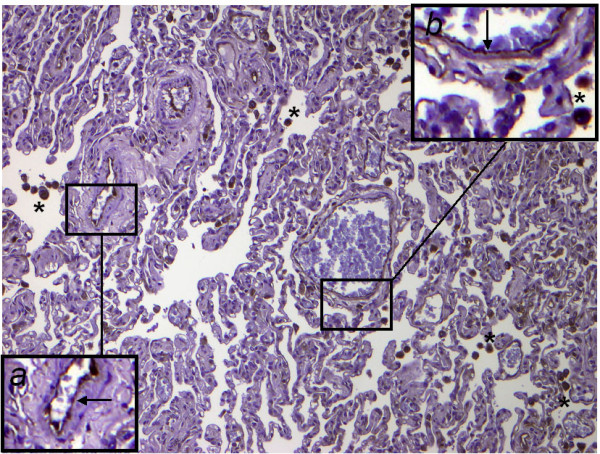
**GDF-15 immunohistochemistry in normal human lung tissue**. Note the staining of endothelial cells in small pulmonary arteries (arrow). Insets depicts a high-power view, highlighting the expression of GDF-15 in endothelial cells. Smooth muscle cells exhibit a weaker signal (arrowhead). Alveolar macrophages show a strong signal for GDF-15 (asterisks). A weak signal for GDF-15 was noted in alveolar and bronchial epithelial cells. Original magnifications: × 100; Inset *a *× 200, inset *b *× 300.

**Figure 3:**
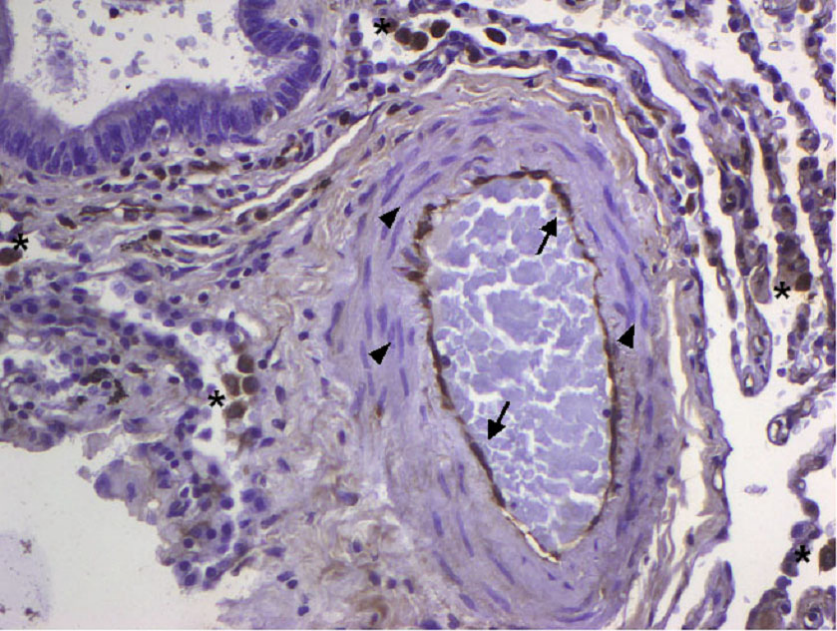
**GDF-15 immunohistochemistry in pulmonary arterial hypertension (PAH)**. GDF-15 protein expression in PAH) showing a strong signal in the endothelial cell layer (arrows) of a pulmonary artery with media hypertrophy. The smooth muscle cells (arrowheads) of the remodeled pulmonary artery are lacking significant GDF-15 protein expression. Macrophages around the pulmonary artery stain positive for GDF-15 (asterisks). Original magnification: × 200.

**Figure 4:**
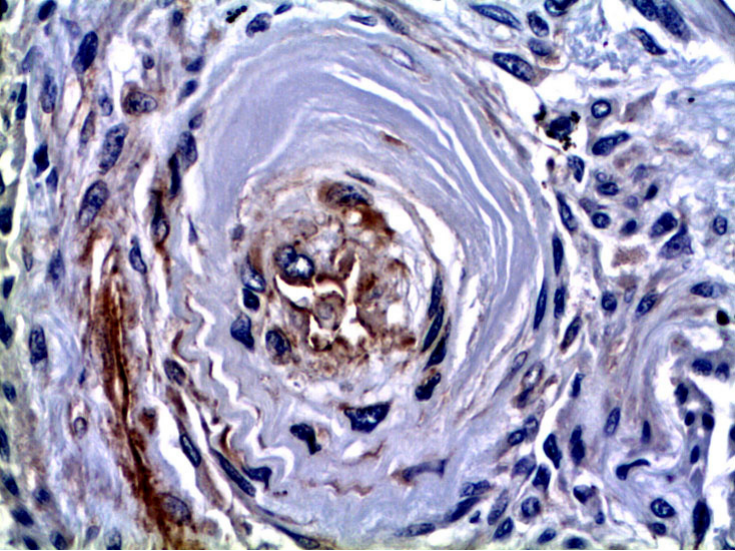
**GDF-15 immunohistochemistry in a concentric lesion of a patient with PAH**. Immunoreactiv-ity for GDF-15 is observed in cells lining the small remaining lumen of the concentric lesion (asterisk). Inset depicts a high-power view of the GDF-15 positive cells, which are probably endothelial cells (arrowheads). Original magnifications: × 200; Inset × 400.

**Figure 5:**
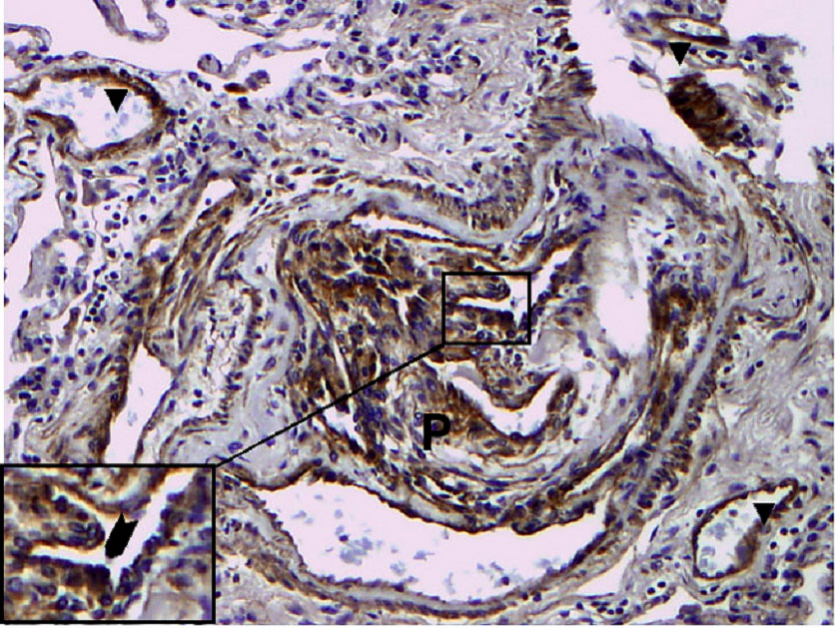
**GDF-15 immunohistochemistry in a plexiform lesion of a patient with IPAH**. Immunohisto-chemical localization of GDF-15 protein in lung tissue of a patient with idiopathic pulmonary arterial hyperten-sion (IPAH). Intense signal for GDF-15 is seen in the cells of a plexiform lesion (P). Inset exhibits prominent luminal staining of GDF-15 in cells lining the vascular channel (arrow). Note the presence of GDF-15 in the endothelial cells of neigbouring small capillaries (arrowheads). Original magnifications: × 200; Inset × 400.

**Figure 6:**
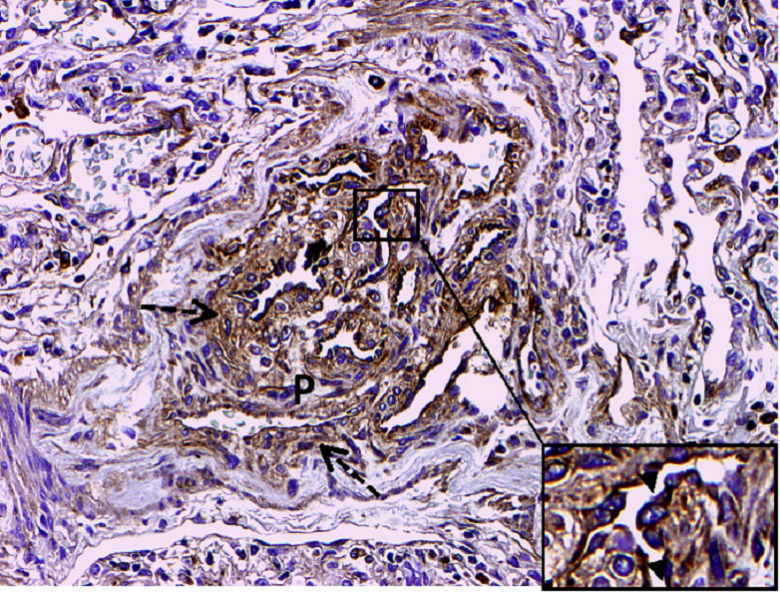
**GDF-15 immunohistochemistry in a patient with PAH and Eisenmenger physiology**. Intense signal for GDF 15 is noted in cells lining vascular channels. Inset shows prominent luminal staining of GDF-15 in endothelial cells (arrowhead). Note lower signal for GDF-15 in the connective tissue around the plexiform lesion, which probably represents GDF-15 bound to extracellular matrix (dashed arrows). Original magnifications: × 200; Inset × 400.

**Figure 7:**
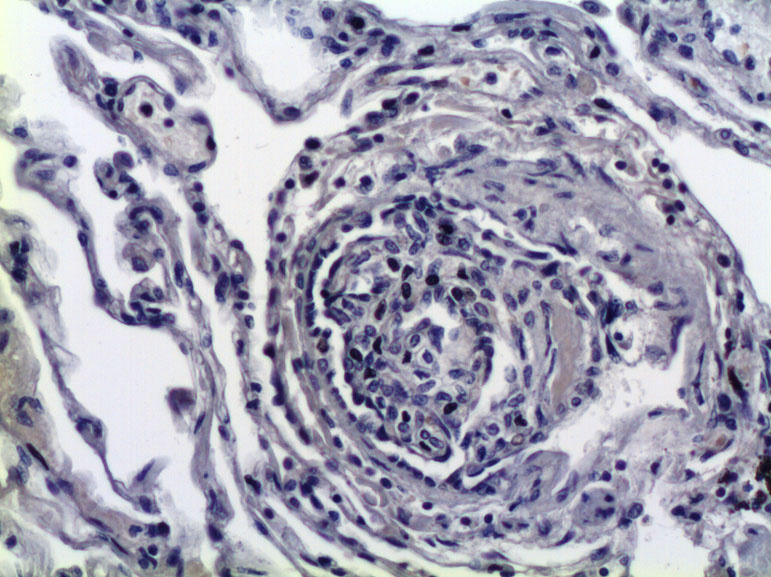
**Negative Control**. Representative photo of a plexiform lesion using a rabbit IgG isotype control for immunohistochemistry. Original magnifications: × 200.

**Figure 8:**
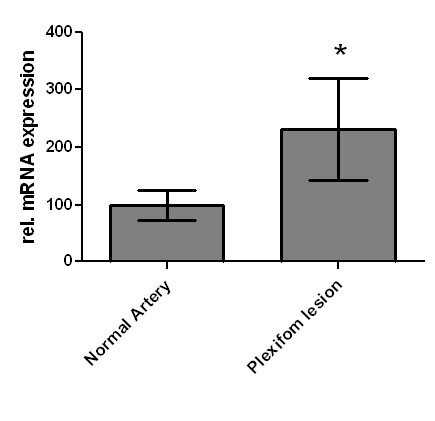
**GDF-15 mRNA expression amplified from laser-assisted microdissection**. Distinct anatomical lung structures (plexiform lesions, normal arteries) of patients with severe PAH were isolated using laser-assisted microdissection techniques. Relative mRNA expression was assessed by real-time RT-PCR. Data are presented as relative expression of GDF-15 mRNA normalized to two housekeeping genes. Data from n = 4 in each group are shown as mean ± SD. * = p < 0.05 vs. normal artery.

GDF-15 mRNA expression in whole lung tissue was assessed using real-time RT-PCR. Com-pared to normal lung tissue, GDF-15 expression was 5-fold increased in lung tissue from PAH patients (Figure 1). To assess protein expression of GDF-15 in human lung we performed immunohistochem-istry studies. In normal lung, GDF-15 was noted in endothelial cells of small pulmonary arteries as well as in alveolar macrophages (Figure 2). Smooth muscle cells and epithelial cells exhibited only a weak signal. In PAH lungs GDF-15 protein expression was observed in the endothelial cell layers of pulmonary arteries with medial hypertrophy, whereas little or no GDF-15 protein expression could be detected in the smooth muscle cells of remodeled pulmonary arteries (Figure 3). In concentric lesions GDF-15 expression was noted in cells lining the small lumen of lesions, probably endothelial cells (Figure 4). In plexiform lesions, an intense signal for GDF-15 protein was observed in the cells lining the vascular channels (Figure 5). There were no differences in the cellular expression pattern of GDF-15 in IPAH (Figure 5) and PAH due to Eisenmenger's physiology (Figure 6). As a negative control we used a rabbit IgG isotype control which was lacking a staining signal (Figure 7). To confirm the GDF-15 expres-sion patterns seen in the immunohistochemistry studies, laser-assisted micro-dissections of vascular compart-ments from normal lungs and PAH lungs were performed (Figure 8). Transcripts for GDF-15 were amplified from laser-captured vascular cells of normal pulmonary arteries and plexiform lesions of PAH patients by using quantitative RT-PCR. Compared to normal pulmonary arteries, a 3-fold increase of GDF-15 transcripts was detected in plexiform lesions of patients with PAH. To study the cellular composition of plexiform lesions, transcripts for the endothelial cell marker CD31 and eNOs as well as the smooth muscle cell marker myosin heavy chain were also amplified from microdissected vascular cells (Additional file 3). Compared to the vessel wall of normal arteries expression of CD31 and eNOS was increased in plexiform lesions. On the other hand, the smooth muscle cell marker myosin heavy chain was also expressed in microdissected cells from plexiform lesions suggesting a heterogenous cellular composition of these vascular structures.

### GDF-15 expression in response to hypoxia and laminar shear stress

**Figure 9:**
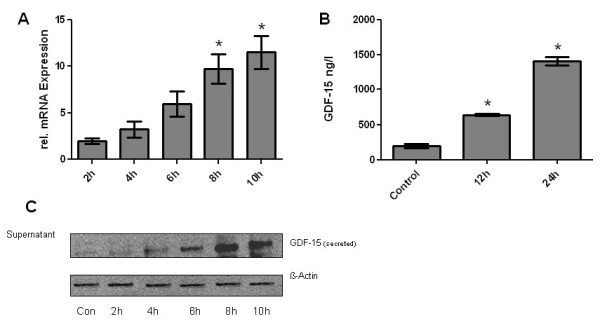
**Upregulation of GDF-15 by hypoxia in endothelial cells**. Human pulmonary microvascular endothelial cells (HPMEC) were subjected to hypoxia for various time periods (2 h to 24 h). The mRNA and protein levels of GDF-15 (secreted form) were determined either by quantitative RT-PCR (panel A), immunoradiometric sandwich assay - IRMA (panel B) or Western Blot analysis (panel C). Hypoxia increased GDF-15 expression in a time dependent manner, which was initially detected after 2 hours on mRNA level and after 4 hours on protein level. Data from n = 4 each group are shown as mean ± SD. *p < 0.05 compared to control.

**Figure 10:**
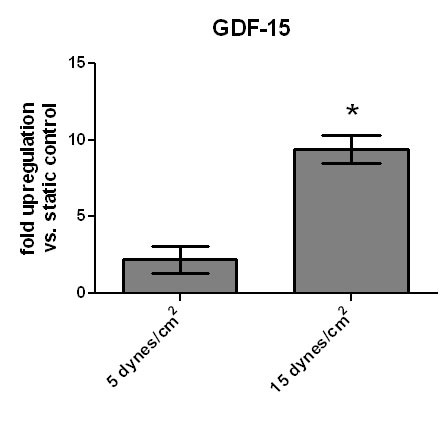
**Upregulation of GDF-15 by shear stress**. Human pulmonary microvascular endothelial cells (HPMEC) were exposed to laminar fluid flow (5 and 15 dynes/cm^2^) for 6 h. Expression of GDF-15 mRNA was assessed by quantitative RT-PCR. Data are presented as relative expression of GDF-15 mRNA normalized to two housekeeping genes (β-GUS and β-actin). Data from n = 5 each group are shown as mean ± SD. * = p < 0.05 compared to static control (0 dynes/cm^2^).

HPMEC were exposed to hypoxia for various time periods. mRNA and protein levels for GDF-15 were determined using quantitative RT-PCR (Figure 9, panel A), IRMA (Figure 9, panel B) and Western Blot analysis (Figure 9, panel C). Hypoxia increased GDF-15 expres-sion in a time-dependent manner, which was initially detected after 2 hours at mRNA level and after 4 hours at protein level. After 10 hours there was a 12-fold upregulation of GDF-15 mRNA. Western Blot analysis from HPMEC exposed to hypoxia showed a strong upregulation of the secreted 30 kDa form of GDF-15. To assess the effects of shear stress on the mRNA expression of GDF-15, HPMEC were exposed to laminar flow (5 and 15 dynes/cm^2^) for 6 h in a cone-and-plate apparatus. Laminar shear stress (5 dynes/cm^2^) resulted in a 2-fold upregulation of GDF-15 transcripts compared to static controls (0 dynes/cm^2^). By increasing the laminar flow to 15 dynes/cm^2^, a 10-fold upregulation of GDF-15 mRNA was noted (Figure 10).

### Effect of GDF-15 on proliferation of pulmonary endothelial cells

**Figure 11:**
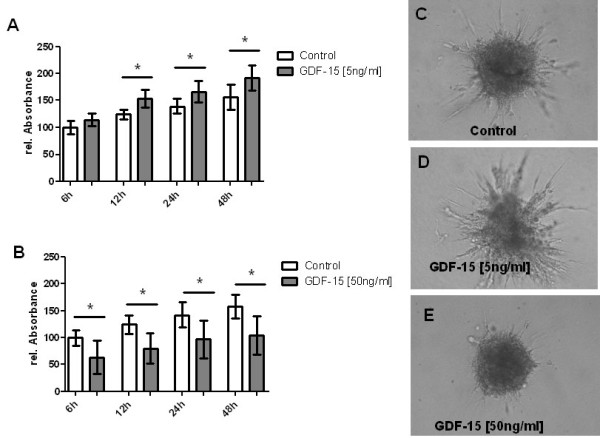
**Effect of GDF-15 on endothelial cell proliferation and sprouting**. Proliferation of human pulmonary microvascular endothelial (HPMEC) cell was assessed using a rapid colorimetric proliferation assay. At a concentration of 5 ng/ml recombinant GDF-15 led to increased HPMEC proliferation (panel A), whereas a reduction of HPMEC proliferation (panel B) was seen at higher concentration of GDF-15 (50 ng/ml). Data from n = 5 each group are shown as mean ± SD. * = p < 0.05 vs. control. Sprouting of human pulmonary microvascular endothelial cells (HPMEC) was assessed using a three-dimensional spheroid sprouting assay. Compared to control (panel C), recombinant GDF-15 protein at a concentration of 5 ng/ml increased endothelial cell sprouting (panel D), whereas at higher concentrations (50 ng/ml) sprouting was decreased (panel E). Five spheroids per group and per experiment were analyzed.

To investigate the angiogenic effects of GDF-15 on HPMEC proliferation, a rapid colorimetric proliferation assay was performed [29]. At a concentration of 5 ng/ml recombinant GDF-15 protein significantly increased endothelial cell proliferation at different time points ranging from 12 h to 48 h (Figure 11, panel A). Whereas 50 ng/ml recombinant GDF-15 incubated for 6 to 48 hours showed a significant inhibition of endothelial cell proliferation (Figure 11, panel B).

### Effect of GDF-15 on sprouting of pulmonary endothelial cells

To investigate the angiogenic effects of GDF-15 sprouting of human pulmonary microvascu-lar endothelial cells (HPMEC) was assessed using a three-dimensional spheroid sprouting assay. Compared to control (Figure 11, panel C), recombinant GDF-15 protein at a concentration of 5 ng/ml increased endothelial cell sprouting (Figure 11, panel D), whereas at higher concentrations (50 ng/ml) sprouting was decreased (Figure 11, panel E).

### GDF-15 affects endothelial cell death in response to hypoxia

**Figure 12:**
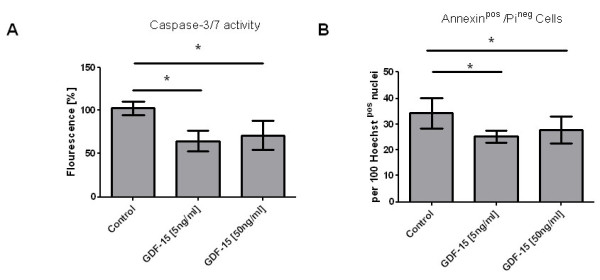
**Effect of GDF-15 on endothelial cell death**. Human pulmonary microvascular endothelial cells (HPMEC) were exposed to hypoxia within an incubator chamber filled with a gas mixture of 0,2% oxygen, 5% carbon dioxide and 94,8% nitrogen placed in a 37°C incubator. Apoptotic cell death was either assessed by measuring the activity of the caspases 3 and 7 (panel A) and by determining the number of Annexin V-positive cells (panel B). Recombinant GDF-15 at a concentration of 5 and 50 ng/ml) reduced hypoxia-induced apoptotic cell death. Data from n = 5 in each group are shown as mean ± SD. * = p < 0.05 compared to control.

**Figure 13:**
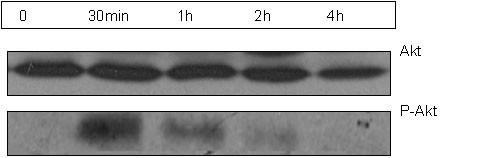
**Akt phosphorylation by GDF-15 in endothelial cells**. GDF-15 induced Akt phosphorylation at Ser437 in human pulmonary microvascular endothelial cells (HPMEC). The cells were stimulated with recombinant GDF-15 protein (50 ng/ml) for 30 to 240 minutes. Akt and Ser437 were determined by immunoblotting. An exemplary blot from n = 3 experiments is presented.

HPMEC were exposed to hypoxia to induce apoptosis. In our hypoxia system the most prom-inent induction of apoptosis was observed after 8-12 hours. Apoptotic cell death was assessed by measuring the activities of caspases 3 and 7 (Figure 12, panel A), two of the key executioners of apoptosis, and by determining the number of Annexin V-positive/propidium iodide-negative cells (Figure 12, panel B). Recombinant GDF-15 protein at a concentration of either 5 or 50 ng/ml reduced hypoxia-induced apoptotic cell death. Stimulating HPMEC with recombinant GDF-15 protein (50 ng/ml) for 30 to 240 minutes resulted in an induction of Akt phosphorylation determined by immunoblotting (Figure 13).

## Discussion

In the present study we demonstrated that GDF-15 is expressed in human lung tissue, arising predominantly in macrophages and pulmonary endothelial cells. Compared to normal lung, GDF-15 appears upregulated in lung tissue of patients with PAH, especially in areas of active vascular remodeling, i.e. plexiform lesions. Since GDF-15 protein influences proliferation and apoptosis of pulmonary endothelial cells, it might play a role in the evolution and homeostasis of plexiform lesions in PAH patients.

GDF-15 is a stress-responsive cytokine that is upregulated under pathologic conditions involving various stimuli such as tissue hypoxia, inflammation, or enhanced oxidative stress [3-6]. Under physiologic conditions GDF-15 is only weakly expressed in most tissues and organs [34]. It is therefore unsurprising that we only detected a weak immunostaining signal for GDF-15 in human normal lung tissue with almost no expression in the airways like bronchial and alveolar epithelial cells. As demonstrated in previous studies [18], GDF-15 was strongly expressed in alveolar macrophages which might indicate a role of this protein in innate immunity [2]. Interestingly, our immunostaining experiments clearly demonstrated strong expression of GDF-15 in the vascular compartment of PAH patients, particularly in the intima of pulmonary arteries. GDF-15 staining was observed in pulmonary vessels of all sizes, beginning from the microvasculature up to large pulmonary vessels. The endothelial expression pattern was observed in normal lung as well as in lungs from PAH patients, suggesting a physiological role for GDF-15 in pulmonary endothelial cells. To date little is known about the functional role of GDF-15 in endothelial cells. A previous study demonstrated inhibitory effects of GDF-15 on proliferation, migration and invasion of endothelial cells *in vitro *as well as anti-angiogenic effects *in vivo *using a matrigel-plug-assay [11]. In contrast to these findings, a recently published paper demonstrated both angiogenic and anti-angiogenic properties of GDF-15 [12], which were concentration-dependent. GDF-15 elicited pro-angiogenic effects at low concentrations, whereas paradoxical effects were observed at higher concentrations (100 ng/ml). In accordance with this finding we too were able to demonstrate concentration-dependent pro- as well as anti-angiogenic effects of recombinant GDF-15 protein on pulmonary endothelial cells *in vitro*. That different concentrations of a cytokine could result in different cellular responses is well-known for members of the TGF-β-family. For instance, TGF-β1 exerts bi-functional effects on endothelial cells, regarding activation, proliferation and migration. At low concentrations TGF-β1 has a stimulating effect, whereas higher concentrations inhibit these processes [35]. It is challenging to speculate the active amount of GDF-15 in the pulmonary vasculature. However, addi-tional autocrine and paracrine pathways may determine the local concentration of GDF-in the vascular compartment. Furthermore, a variety of activating or disabling regulators may interfere with the intra- and extracellular storage as well as the stability of GDF-15 in lung compartments.

Compared to normal lung tissue, increased GDF-15 expression was observed in PAH lungs, with strongest expression being identified in areas of vascular remodeling, especially in the cells forming the plexiform lesions. In comparison, GDF-15 expression was lower in vascular smooth muscle cells, both in normal vessels and in remodeled arterioles with media hypertrophy. No differences in the expression pattern of GDF-15 were seen between lungs of various underlying aetiologies of pulmonary hypertension such as IPAH, and PAH due to Eisenmenger's physiology. A recent study identified expression of GDF-15 protein in pulmonary macrophages of patients with PAH due to scleroderma, but almost no GDF-15 staining in IPAH lungs [18]. This staining pattern appears to conflict with our results, but may be related to different protocols of tissue preparation and staining. To confirm the expression pattern seen in our immunohistochemical studies we performed laser-assisted microdissection of vascular subcompartments in PAH lungs. We successfully amplified GDF-15 transcripts in plexiform lesions and cells from morphological normal pulmonary arteries of PAH patients. In accordance to the immunohistochemical staining pattern, increased GDF-15 expression was detected in plexiform lesions compared to unremodeled pulmonary arteries. These findings suggest that GDF-15 could be involved in the pathobiology of plexiform lesions as opposed to the muscular compartment. The cellular and cytokine environment of plexiform lesions, which are characterized by disorganized focal proliferation of endothelial channels [36,37], is complex and not fully understood. Since a variety of different cytokines and signaling pathways interact with each other, it is difficult to define the precise role of a single cytokine in such a complex milieu. Key players in vascular remodeling of PAH lungs are members of the TGF-β-superfamily, and TGFβ1 has been reported to potentiate intimal hyperplasia in animal models following arterial injury [38].

Factors triggering expression of GDF-15 in the pulmonary vasculature remain unclear. Since GDF-15 is a stress responsive cytokine speculation remains that inflammation and oxidative stress trigger expression of GDF-15 in plexiform lesions. Indeed, several studies have demonstrated increased oxidative stress and inflammation within plexiform lesions [39]. Our findings indicate that hypoxia is a potent stimulator of GDF-15 expression in pulmonary endothelial cells. Furthermore shear stress might lead to induction of GDF-15 expression in the pulmonary vasculature. Given that in severe PAH, plexiform lesions tend to form at bifur-cations [40] where shear stress is likely to be high, we examined whether shear stress affects GDF-15 expression. We were able to demonstrate that shear stress leads to an upregulation of GDF-15 expression in human microvascular endothelial cells. These findings may be significant, regarding the evolution of an apoptosis-resistant endothelial cell phenotype. Previous reports have shown that shear stress has an anti-apoptotic effect on endothelial cells [41]. Since shear stress is a potent inducer of GDF-15 in endothelial cells it is possible that the anti-apoptotic effect provoked by shear stress is - at least partly - mediated by GDF-15. In our study we were able to demonstrate that GDF-15 caused an induction of Akt phosphorylation and had a prosurvival effect on endothelial cells. This finding is in accordance with documented anti-apoptotic effects of GDF-15 in cardiomyocytes involving the phosphoinositide 3-OH kinase (PI3K) and Akt-dependent signaling pathways [32]. The net effect of GDF-15 on cell proliferation, apoptosis and pulmonary vascular remodeling is difficult to evaluate, especially as GDF-15 is not the only player among the mediators orchestrating vascular remodeling. Like other members of the TGF-β-family proteins, GDF-15 executes a wide variety of complex and ambiguous functions, depending on cell type, microenvironment and genetic status of the cell.

## Conclusions

In conclusion, GDF-15 is up-regulated in lungs from patients with PAH where it is mainly located in vascular endothelial cells and plexiform lesions. The induction of GDF-15 expression by shear stress and hypoxia in combination with its effects on cell proliferation and apoptosis suggests a functional role of this protein in pulmonary endothelial cells and thereby in the pathobiology of complex vascular lesions in PAH lungs.

## Competing interests

The authors declare that they have no competing interests.

## Authors' contributions

NN and DJ planned the concept and study design. HAG coordinated the study and drafted the manuscript. LM and JR carried out the immunohistochemistry and real time PCR. CS and NN performed the cell culture experiments. CB and LM carried out the laser-assisted microdissection experiments. TK performed the GDF-15 Sandwich IRMA. FL and UL made substantial contributions to the analysis and interpretation of the data. TW and MMH participated in the design of the study. MG critically read and corrected the manuscript. All authors read and approved the final manuscript.

## Supplementary Material

Additional file 1 **GDF-15 immunohistochemistry in human placenta and prostate cancer**. GDF-15 protein expression (brown staining) assessed by immunohistochemistry in normal placental tissue (panel A) and prostate cancer tissue (panel B). Original magnifications: × 100. Click here for file

Additional file 2 **GDF-15 immunohistochemistry using a Goat anti-human GDF-15 IgG antibody**. Immunohistochemical localization of GDF-15 protein in lung tissue of a patient with idiopathic pulmonary arterial hypertension (IPAH) using Goat anti-human GDF-15 IgG antibody (R&D Systems). A signal for GDF-15 was seen in macrophages and cells of a plexiform lesion. Original magnifications: × 200. Click here for file

Additional file 3 **Expression of endothelial cell and smooth muscle cell marker in plexiform lesions**. Distinct anatomical lung structures (plexiform lesions, normal arteries) of patients with severe PAH were isolated using laser-assisted microdissection techniques. Relative mRNA expression was assessed by real-time RT-PCR. Data are presented as relative expression of CD31, eNOS and myosin heay chain mRNA normalized to two housekeeping genes. Data from n = 4 in each group are shown as mean ± SD. * = p < 0.05 vs. normal artery. Click here for file
