# Supplementary figures and images for: GDF-15 is abundantly expressed in plexiform lesions in patients with pulmonary arterial hypertension and affects proliferation and apoptosis of pulmonary endothelial cells
Source: Respir Res. 2011 May 6;12(1):62. doi: 10.1186/1465-9921-12-62 (PMC3113721; doi:10.1186/1465-9921-12-62)

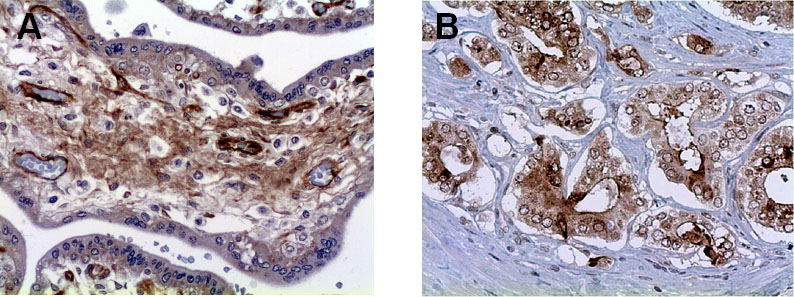

Supplement: Additional file 1 — GDF-15 immunohistochemistry in human placenta and prostate cancer. GDF-15 protein expression (brown staining) assessed by immunohistochemistry in normal placental tissue (panel A) and prostate cancer tissue (panel B). Original magnifications: × 100. [file 1465-9921-12-62-S1.JPEG]

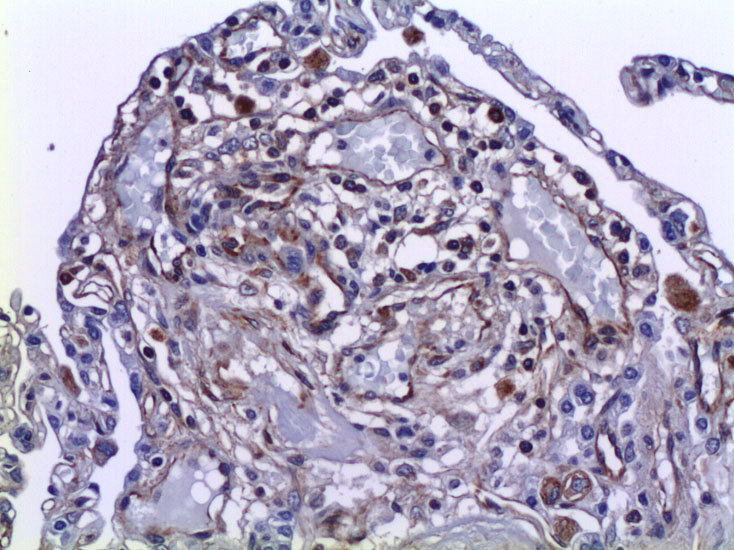

Supplement: Additional file 2 — GDF-15 immunohistochemistry using a Goat anti-human GDF-15 IgG antibody. Immunohistochemical localization of GDF-15 protein in lung tissue of a patient with idiopathic pulmonary arterial hypertension (IPAH) using Goat anti-human GDF-15 IgG antibody (R&D Systems). A signal for GDF-15 was seen in macrophages and cells of a plexiform lesion. Original magnifications: × 200. [file 1465-9921-12-62-S2.JPEG]

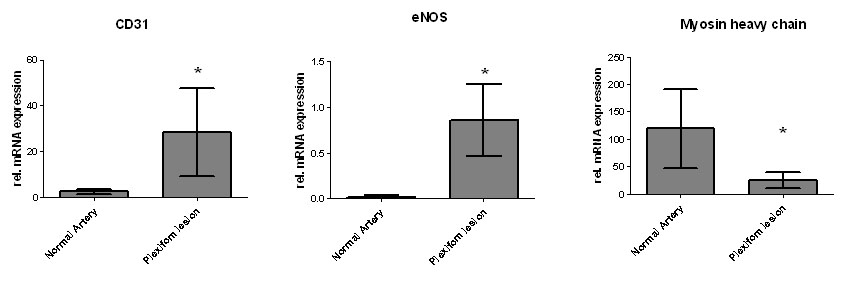

Supplement: Additional file 3 — Expression of endothelial cell and smooth muscle cell marker in plexiform lesions. Distinct anatomical lung structures (plexiform lesions, normal arteries) of patients with severe PAH were isolated using laser-assisted microdissection techniques. Relative mRNA expression was assessed by real-time RT-PCR. Data are presented as relative expression of CD31, eNOS and myosin heay chain mRNA normalized to two housekeeping genes. Data from n = 4 in each group are shown as mean ± SD. * = p < 0.05 vs. normal artery. [file 1465-9921-12-62-S3.JPEG]
